# Exploring a sustainable building’s impact on occupant mental health and cognitive function in a virtual environment

**DOI:** 10.1038/s41598-021-85210-9

**Published:** 2021-03-11

**Authors:** Ming Hu, Madlen Simon, Spencer Fix, Anthony A. Vivino, Edward Bernat

**Affiliations:** 1 School of Architecture, Planning and Preservation, University of Maryland, 3835 Campus Drive, College Park, MD 20742 USA; 2 Department of Psychology, University of Maryland, College Park, MD USA

**Keywords:** Neurophysiology, Environmental impact, Psychology and behaviour, Sustainability, Cognitive neuroscience, Visual system

## Abstract

Even though people spend the majority of their time indoors, the role of buildings in shaping human experience is still not well understood. The objective of this experimental project is to develop, test, and validate a data-driven neuroscience approach to understand the built environment’s impact on occupant cognitive function and mental health. The present study utilized virtual environments and electroencephalogram (EEG) and event-related potential (ERP) approaches, to provide objective neurophysiological information about how sustainable buildings (SBs) impact people’s affective and cognitive functioning differently compared to conventional building (CBs). The long-term goal is to assess the validity of sustainable building design protocols in promoting and increasing mental health and well-being and the mechanism used to accomplish these increases. The findings showed test subjects demonstrated increased visual system engagement and modulated attentional focus and control processing in the SB compared to the CB environments. The findings can be explained by the cognitive load theory, which is consistent with the interpretation of greater focus on the present environment and reduced internal mental processing (cf. mindfulness), based on the observed increased theta/delta activities and greater engagement of visual systems and corresponding decreases in frontal activity in the SB environment. In addition, the combination of virtual environment (VE) and EEG/ERP has the potential to advance design methods by soliciting occupants’ responses prior to completion of the projects. Building design is more than aesthetics; expanding the horizon for neuroscience would eventually result in a new knowledge base for building design, particularly sustainable building design, since the sustainability of the building often needs to be quantified.

## Introduction

Given that Americans spend about 87% of their time inside buildings, the quality and design of buildings are important contributors to human well-being^1^. The trend in sustainable building (SB) models, catalyzed by the founding of the Green Building Council in 1993, offers a unique opportunity to leverage not just beneficial environmental effects but also occupancy well-being effects in the design and construction of new buildings. Case studies have consistently demonstrated the potential for sustainable buildings to increase “soft” benefits of improved well-being and productivity via surveys (self-reported assessments). Current building impact evaluation tools that measure occupants’ well-being and cognitive functions are user response surveys, such as a health and work performance questionnaire and various building wellness surveys. Surveys have two main weaknesses. First, as there are many variables affecting an occupant’s response to the built environment, such as familiarity with the space, time of day when the survey is conducted, and the ambient condition of the environment (e.g., temperature, smell, noise), confounding non-design factors can be hard to disentangle, to control for, and to interpret. Second, the survey response is an indirect measure of the environment, reliant on the user’s opinions (perceived likes and dislikes) and cannot provide objective data about particular environments and features. What is needed are consistent, reliable, and physiologically based measures of mental health effects that capture human response to discrete architectural elements—especially in the pre-build design phase. In order to have such a reliable measurement, an innovative approach is needed.

Substantial evidence now indicates that exposure to natural environments provides health benefits, including stress reduction as well as an increased positive attitude towards resource conservation^2,3^. Sustainable building design has focused on incorporating external views of nature, appropriate internal spatial dimensions and visual connections, and key aspects of resource conservation. However, there has been little empirical work to assess the impact of these design features on occupant stress, behaviors, and attitudes. The perspective taken in this work, consistent with emerging literature, is that SBs may convey benefits similar to nature exposure, and that these benefits can be understood relative to a mindfulness framework^4,5^. We employ a narrow definition of mindfulness in this work, meaning a greater focus on the present moment through greater engagement with the built environment and activities occurring in it. This is consistent with the core aspects of mindfulness as defined across current theories^1–3^.

The objective of this experimental project is to develop, test, and validate a data-driven neuroscience approach. The present study utilized virtual environments and electroencephalogram (EEG) and event-related potential (ERP) approaches, to provide objective neurophysiological information about how sustainable built environments impact affective and cognitive functioning in building occupants. The goal is to assess the validity of sustainable building design protocols in promoting and increasing mental health and well-being and the mechanisms used to accomplish these increases. To this extent, there were two hypotheses tested using the combined virtual environment and EEG/ERP approach:

### Hypothesis 1

Compared to CBs, occupants will respond to SBs with higher engagement, particularly to the sustainable visual stimuli; hence, SBs are associated with **increased visual system engagement** compared to CBs.

### Hypothesis 2

Compared to CBs, occupants of SBs will exhibit modulated attentional focus and control processing.

## Existing research and gap

Cognitive neuroscience is the scientific field studying neural activities which are involved in mental process and their connection to human behavior. It was proposed by psychologists in 1976. As a research approach, its adoption in studying design in built environment is relatively recent, and it presents a promising method for the study of human response to the design of the built environment. Traditionally, in design-related disciplines, there is lack of quantitative methods and tools to study human’s response. Such gap is particularly present in large-scale realistic settings, including buildings and building blocks. Before the emergence of advanced neuroscientific research devices, including functional magnetic resonance imaging (fMRI) and electroencephalography (EEG), implementing cognitive neuroscience research approach in built environment has been difficult. Compared to fMRI that picks up brain signal relying on blood flow, EEG has better temporal resolution since EEG signals are the result of the synchronous activity of neuronal assemblies that can be recorded from the surface of the scalp and are non-invasive^6^.

EEG is often used to measure event-related potentials (ERPs). In the built environment the event can be defined as views, color, texture, light and special spatial features. ERPs has the ability to measure continuous stimulus in the built environment and their related responses, which is very appropriate for studying the human’s response when immersed in a built environment. Meanwhile, built environment is a complex system, continuous measurement can reveal more evidence and provide more clues on which events (stimuli) are induced by which particular design features. There are a few studies exploring application of EEG/ERP in built environment. Ulrich^7^ using EEG signal along the heart rate to examine people’s response to different built environment, he found the difference in response to urban and rural environments^7^. Nguyen and Zeng (2010) used the EEG signals and heart rate variability on seven subjects to study their mental stress when performing design tasks. The data analysis showed the highest stress level was linked to lowest cognitive function, and the cognitive function was related to creativity. The potential conclusion was the lower stress level and higher cognitive function can result in a higher potential for creativity^8^. In a more recent study, Hu et al.^9^ applied EEG to predicate the receptance towards the design outcomes, their study established a basis to connect cognitive engagement to the engineer design outcomes^9^.

The application of EEG to study the built environment’s impact on humans has been focusing on emotion and well-being at large urban levels, because of the link between spatial cognition and underlying neural responses^10^, and on the existing (built) environment. Aspinall et al. (2013) used an affordable EEG device called the Emotiv EPOC to study participants who took a 25-min walk through three areas of Edinburgh, Scotland. Their study findings showed a high-dimensional correlation between green space and emotional change. To be more specific, they found when people move into a green space from a dense urban block, the test subjects showed positive emotion, such as lower frustration, higher engagement, higher interest (arousal) and calmness. Another research team at the University of Edinburgh, Roe et al. (2013), used the same EEG device (Emotiv EPOC) to study 20 participants by showing them a series of urban versus landscape scenes based on the attention restorative theory proposed by Rachel and Stephen Kaplan in 1989^11^. While the mobile EEG integration in large urban-scale research to study emotion is increasing , studies using EEG/ERP on a building scale are still lagging behind. Also, applying EEG/ERP to study the cognitive effect from stimulus in building to occupant is limited. Most recently, a few studies utilizing EEG measure the impact of indoor thermal environment (temperature, humidity) on human’s cognitive function^12–14^. The cognitive functions studies include semantic thinking, visual perception, spatial reasoning and spatial working memory^15^. Li et al.^15^ studied three different types of space in virtual reality: open natural environment, semi-open library environment and closed basement space, and measured the changes in EEG signal data while the experiment subjects were asked to complete a cognitive test. They found when the physical environment factors were changed, people’s work efficiency changed and associated with the occupant satisfaction level of spatial perception^15^. But they did not provide detailed explanation and evidence on how those vastly different environment parameters affected people’s spatial perception. There is great need to examine other built environment parameters, especially visual stimulus, such as light, color, views. To fill the gap, this study focused on the visual stimulus impact on building occupant’s cognitive function. In addition, very little research has looked into how to integrate ERPs into experimental design protocols and use event-related responses to evaluate design options. Therefore, the combination of virtual environment (VE) and EEG/ERP has the potential to advance design methods by soliciting occupants’ responses prior to completion of the projects.

## Theoretic foundation and methodological framework

### Theory and background

**Figure 1:**
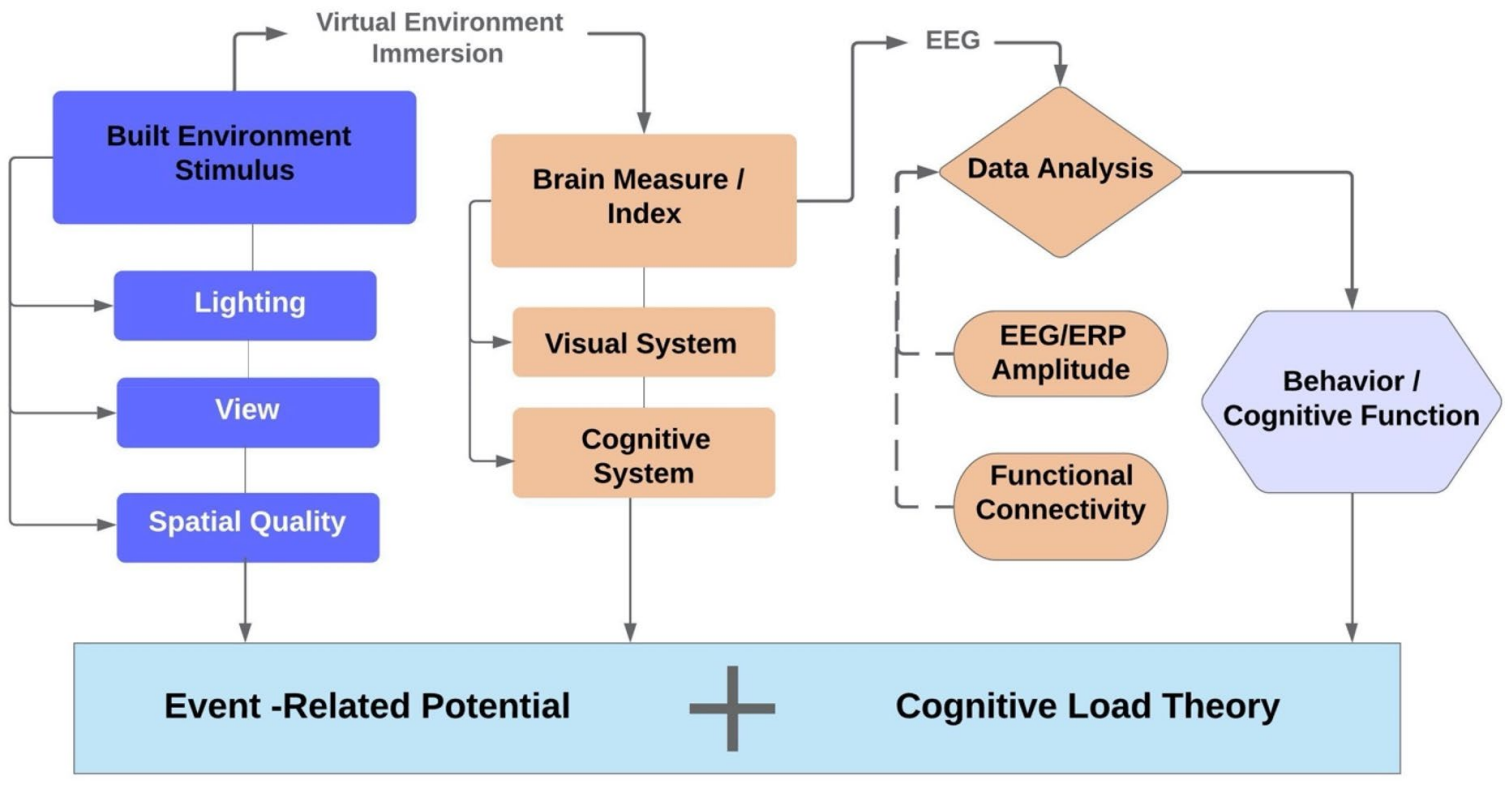
Theoretic foundation and methodological framework based on ERP and CA using a combination of VR and EEG.

Our theoretic foundation and methodological framework is based on the combination of an EEG/ERP neuroscience approach and cognitive load theory (CLT) (see Fig. 1). ERPs are very small voltages generated in the brain structures in response to specific events (stimuli)^16^; ERPs occur or are absent during an event. An event is a time period of interest, and in a visual environment, an event could be exposure to different visual stimuli, such as an image, a word, a sign, a tree, or light from outside. An EEG device is used to measure ERPs that demonstrate brain activity directly related to a specific stimulus. The continuous EEG/ERPs are particularly appropriate for assessing a building’s impact on humans, since an occupant’s experience, perception, and response to a building is the accumulation of that person’s entire experience inside of the building. A building is composed of multiple spaces, so the continuous measurement of brain activities could provide us with more accurate insight of a building’s impact. CLT was initially developed by John Sweller while he was studying problem-solving in 1988^17^; other researchers built upon his theory and further developed the CLT model. CLT is a theoretical framework based on previous knowledge of human cognitive architecture in the brain^18^, which includes long-term memory and working memory. CLT comprises two types of cognitive loads: intrinsic load and extrinsic load^19^, with intrinsic load defined by the nature of the task itself, and extrinsic load determined by the way in which the task is presented^14^, including in which type of environment the task is presented. Plass and Van Merriënboer proposed a CLT model in 1994^20^ and revised it in 2014 to include a “physical learning environment,” which is disentangled from “learning tasks” and “learning environment” in order to describe the physical characteristics of the built environment in which cognitive tasks (such as learning) happen^15,16^. They recognized the importance of studying the causal effect of the physical built environment on cognitive load based on their research findings on impacts of the physical environment on behavior, performance, and attitude in a learning environment^21,22^. According to Plass and Van Merriënboer, the physical environment characteristics include volume, density, lighting, spatial arrangement, and the presence of other people, and it is not easy in research to distinguish those physical learning environments from the learning task itself^15^. Recently developed novel technology, such as VE, is useful to help in disentangling those variables.

Figure 1 illustrates the theoretic foundation and methodological framework of this project. In this study, we first manipulate three specific built environment stimuli (listed in blue color) to induce the brain activity (ERP) in SBs and CBs. During the VE immersion, we collected data on visual and cognitive system responses using EEG device (listed in orange color). Then we utilized the collected data to study the Time–frequency and Functional Connectivity (explained in the findings), in order to further our understanding of built environment’s impact on occupant’s behavior and cognitive function.

### Built environment parameters

In this study, we used three sustainable built environment parameters as the design basis for testing our hypotheses: lighting, view, and spatial arrangement. All three characteristics are required design elements in the most commonly accepted and utilized “green building” design guidelines and rating systems, namely LEED, WELL, and the Living Building Challenge. Appendix A lists the requirements extracted from those design guidelines for the three parameters.

#### Lighting

The primary characteristics of lighting shown to influence short-term psychological moods include light levels, light color, spectral distribution, and temporal patterns^23–25^. Long-term psychological and physiological effects from non-visual aspects of light emerged from circadian rhythm research^24^. Both artificial lighting systems and access to daylight have large impacts on building occupants’ circadian systems for two reasons. First, as the circadian system fundamentally underpins human physiology, when disrupted, extensive ripple effects are likely to occur. Second, as people spend the majority of their time indoors, it is highly likely that the circadian system is affected by indoor lighting conditions. Studies have suggested that some interior light levels are insufficient to appropriately regulate the circadian clock. Under such conditions, “biological darkness” and attendant effects predominate, including fatigue and excessive sleepiness^24^. In the last decade, a large body of research has been published due to the interest in lighting and people’s well-being and productivity. The consensus is that ambient light and its physical characteristics are major modulators of brain function and cognition^24^.

#### View

Views are directly related to the concept of biophilic design, coined by Edward. O. Wilson in 1984, which refers to the basic human need to affiliate with life and lifelike processes^26^. Pioneering environmental psychologists Rachel and Stephen Kaplan^2^ proposed the attention restoration theory, which states that nature has the ability to restore people’s attention and release stress^27^.They demonstrated that people tend to prefer natural environments over built environments, and built environments with water, trees, and other vegetation more than built environments without such features^28^. Researchers continue to explore how building elements that represent, mimic, or provide access to nature environments impact restorative functions and well-being. Building views can be categorized into three types. The first is a direct visual connection to the outdoor natural environment^29^. An early study conducted by Roger Ulrich at Pennsylvania hospitals found that surgical patients assigned to rooms with windows looking out on a natural scene had shorter postoperative hospital stays^30^. The same restorative effects have been shown in other settings, including the home^28^, school^31^, and office^32^. The second type is the visual connection to indoor natural elements, such as a green wall or water feature. In 2016, a research team studied classrooms in two elementary schools for four months. The results showed that children scored better on a test for selective attention when taken in classrooms with green walls; there was no difference in the children’s self-reports on well-being^33^. The third type of view is that of other people’s life activities. Therefore, a view—either of nature or green elements within buildings, or of other living forms (such as other occupants)—is an excellent and flexible design parameter for this research.

#### Spatial arrangement

Spatial arrangement and its impact on productivity and cognitive functioning belongs within the field of environmental psychology. Studies that investigated the impact of a building’s interior layout (floor plan) have had varied results. Early studies by Hedge^34^ and Sundstrom et al. (1980)^35^ found worker dissatisfaction to be correlated with increased spatial openness. Brennan et al.^36^ conducted a longitudinal field study to examine workers’ satisfaction and productivity before and after relocation from traditional (enclosed) offices to open offices. They found that workers were less satisfied, in terms of increased disturbances and decreased privacy, following the move,the dissatisfaction persisted even after an adjustment period^36^. Other research showed that layout-scale spatial measures are better predictors of how well occupants rate the capacity of a work environment to support collaboration, in comparison with workstation-scale measures^37^. A U.S. national survey study in 2003 demonstrated that physically attractive school environments (middle and high schools) were associated with less problematic and risky student behaviors, whereas the less physically attractive environments were not^38^. However, this study only differentiated attractiveness from cleanness, and did not define attractiveness. Subsequent researchers have postulated that a school’s multi-dimensional spatial qualities (physical environment design) are imbued with different meanings and messages for students. An attractive, clean, and orderly space indicates a high spatial quality and conveys to students the message that this is a place where learning and growth are both valued and supported^40^. But, in general, few studies have explored this area, as spatial characteristics are difficult to define and measure.

## Method and materials

### Experimental stimuli: simulated virtual environment

**Figure 2:**
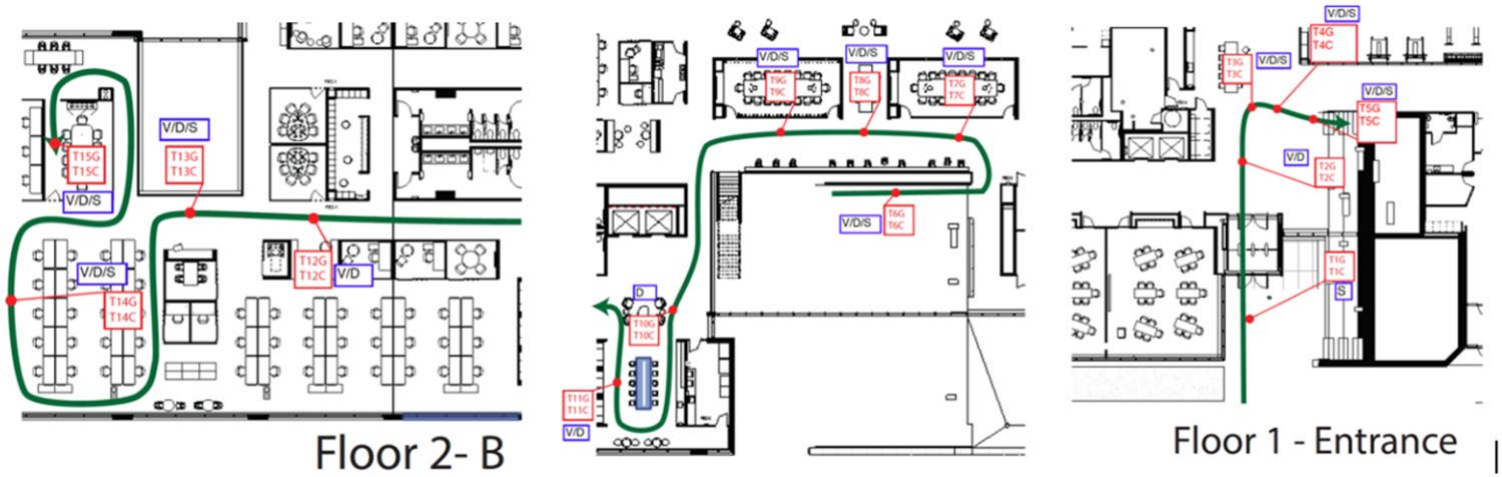
Walk-through routine in virtual environment.

In 2018 and 2019, the research team conducted experiments using the three SB parameters (lighting, view, spatial arrangement) with 36 participants. Two different models of the same three-dimensional virtual building were built by using Autodesk Revit software to construct SB and CB designs. These were then rendered using the plug-in virtual reality tool, Enscape, into two “real time” virtual buildings/designs for use in the experiment. The simulated environment consisted of a two-story building composed of four different spaces. In the SF, these spaces were, (1) public: an entry lobby and open staircase, (2) semi-public: a collaboration space, a conference room, and an open kitchen; (3) semi-private: a fitness center and a conference room in an open office; (4) private: an individual working space. When the participants were in the virtual environment, a “preset walk-through” allowed them to get comfortable with the equipment and the experience. Figure 2 illustrates the floor plan with a walk-through route, and the locations where the participants stopped and looked around in the virtual environment. These stop locations were preset. Participants first entered through the building lobby, then walked up to the second floor’s open office area through a large open staircase. During the two-floor tour, they encountered the fitness center, the open kitchen, the collaboration space, and conference rooms.

**Figure 3:**
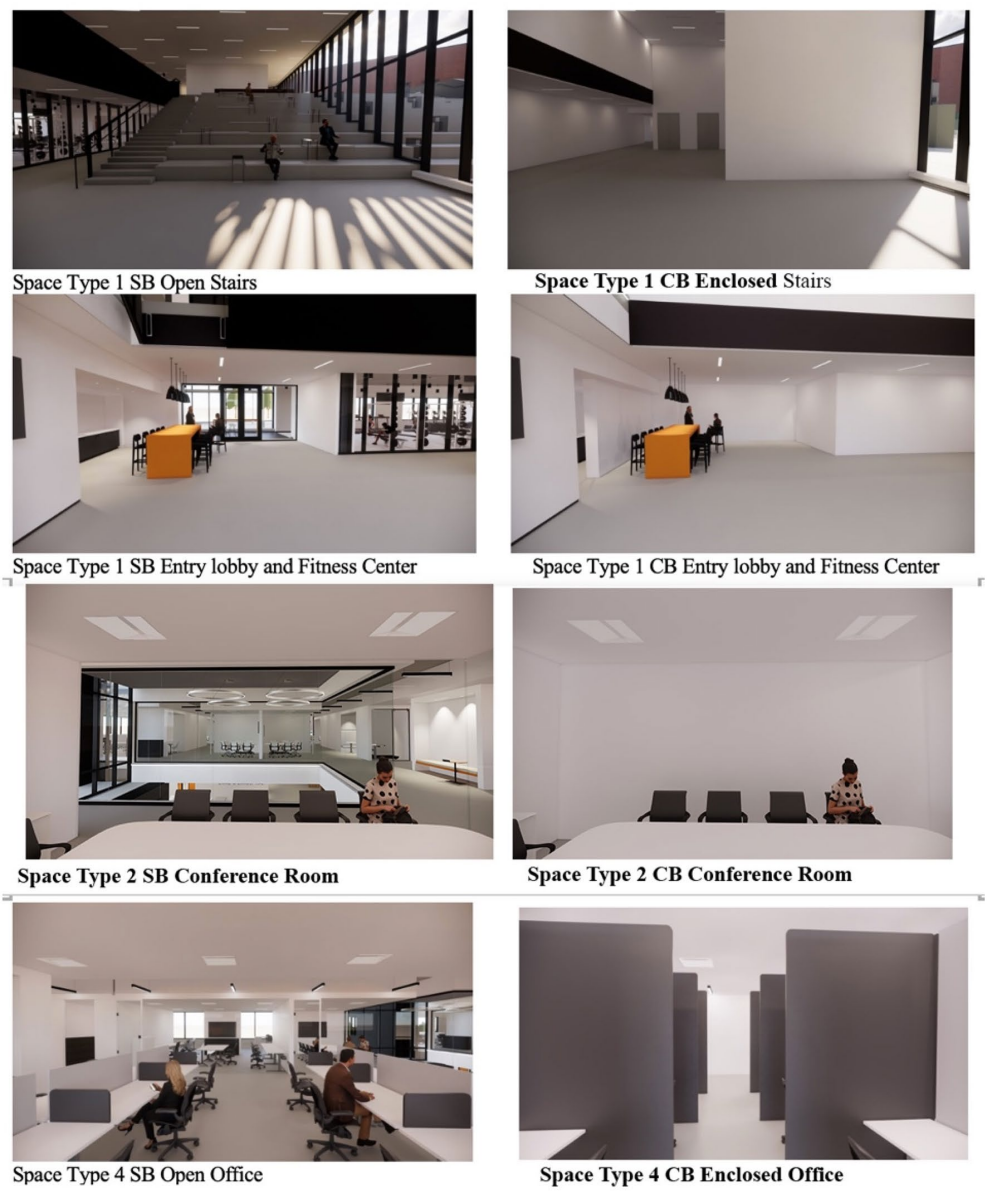
Virtual environments of the SB and CB designs.

Three major changes were made in the CB design to differentiate it from the SB environment: (i) public space: the large open staircase in the lobby was replaced by two conventional elevators and a small enclosed staircase; (ii) semi-public: all open breakout spaces, the kitchen, and the collaboration area were enclosed by solid walls to block the views into the semi-public spaces; (iii) semi-private: the glass walls of the conference and fitness center were replaced with solid dry wall to block natural daylight and the view to the outside; (iv) private: the open floor working area was replaced by individual cubicles with high partition walls, removing the visual and verbal connections between different working stations. The differences in the four space types between the SB environment and control CB environment are illustrated in Fig. 3.

### Participants

Thirty-six undergraduate students were recruited through the psychology subject pool or the community, with each receiving either course credit or fifty dollars for their participation. They were recruited between August 2019 and February 2020. Nine of the participants were women. The age range was 17–23 years, with a mean of 18.41 (S.D. = 1.28), and all reported no history of neurological or mental abnormalities. The study protocol was reviewed and approved by the Institutional Review Board of the University of Maryland. All methods were performed in accordance with the relevant guidelines and regulations. For participants under the age of 18 years, informed consent was obtained from parents and/or legal guardian for both study participation and publication. The informed consent was obtained from the participants (above 18 years of age) for both experiment and publication.

### Experiment procedure and engagement tasks

**Figure 4:**
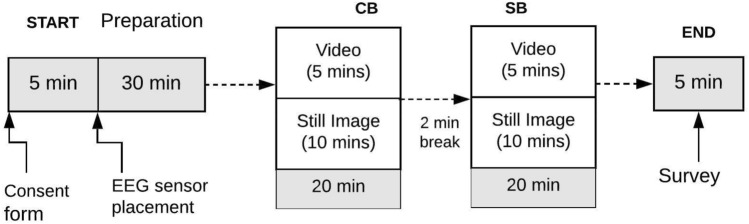
Experiment procedure.

Figure 4 shows the two-step experiment procedure that was developed for measuring experiences of both buildings. To allow utilization of both continuous and event-related EEG/ERP analytic methods, for which there is a large research base to draw inferences from, the present study employed both still images (event-related) and movies (continuous). In total, 45 still images and a five-minute video were created for the SB and CB designs, respectively. The movie was presented first, to familiarize the participant with the built environment. The movie followed the path detailed in Sect. 4.1 (Experimental stimuli: simulated VR environment). Participants were asked to simply view the building as they were moved through the building’s environment. Analytic approaches were aggregated across the entire movie. Next, we presented still images from the movie of key points along the path (see Fig. 2). Analytic approaches focused on event-related (i.e., ERP) approaches to infer activity. Test subjects spent 25 min in each building. With sensor placements, task practice, and acclimation to the virtual viewing, one subject testing took less than 1.5 h.

**Figure 5:**
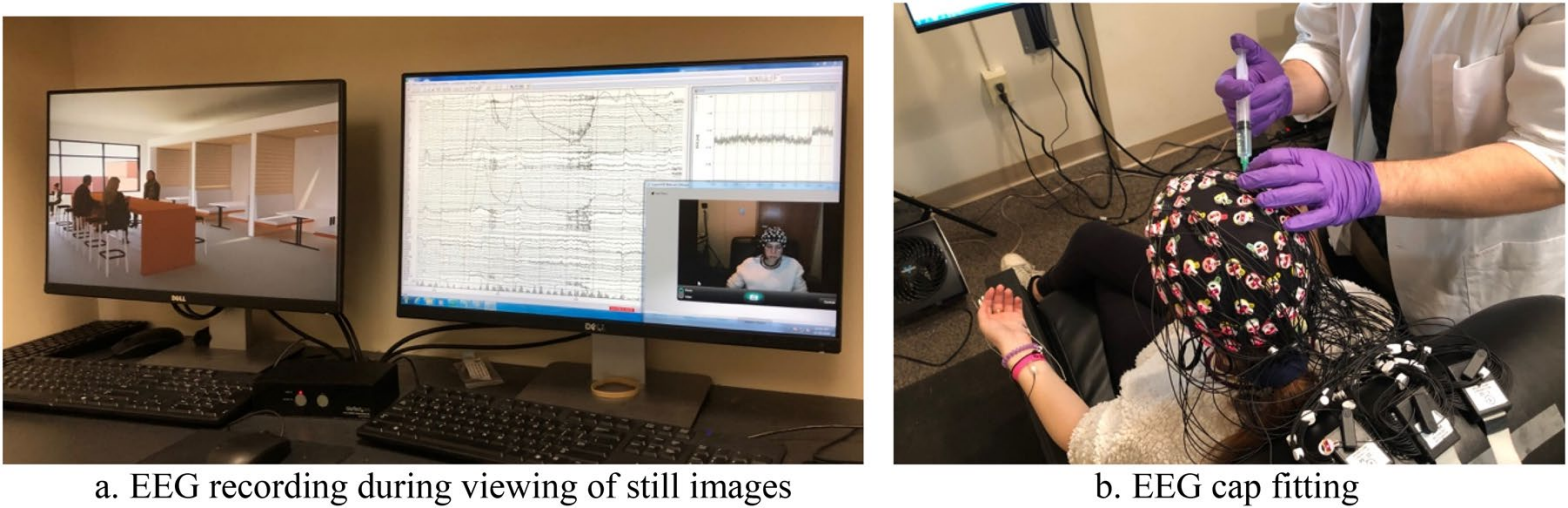
(**a**) EEG recording during viewing of still images. (**b**) EEG cap fitting.

Figure 5a shows how the EEG/ERP data was recorded while testing subjects were viewing the video and still images. Figure 5b illustrates the testing conditions: test subjects wore an EEG cap (104-channel (96-ch EEG) BrainProducts Actichamp active electrode systems).

### Data collection and preprocessing

#### Stimulus presentation

Data collection was conducted in a dimly lit, sound-attenuated room. Experimental stimuli were presented on a 24-inch Dell high-definition LED color monitor, centrally placed at a viewing distance of 100 cm, using E-Prime version 2.0.

#### Psychophysiological data collection

Data was recorded using a BrainVision 96-channel actiCAP (sintered Ag–Ag/Cl; 10–20 layout) as well as a 96-channel actiCHamp amplifier (EASYCAP GmbH). Horizontal electrooculogram activity was recorded from electrodes on the outer canthus of both eyes, while vertical electrooculogram activity was recorded from electrodes placed above and below the left eye. Impedances were kept below 15 kΩ. EEG signals were vertex referenced during the recording and digitized at 500 Hz using the BrainVision Pycorder (Brain Products GmbH).

#### Data preprocessing

Data preprocessing followed similar work from our group^39,40^. To summarize this procedure, epochs of 1000 ms pre- to 2000 ms post-picture onset were extracted with a 150 to 10 ms pre-picture baseline and were referenced using averaged mastoid sites. Ocular artifacts were corrected using a regression-based algorithim by Gratton, implemented in EEGLAB^41,42^. Then, two methods of data cleaning were used. In the first method, trials were rejected if activity at F3 or F4 exceeded ± 100 µV in either the pre-stimulus period of -1000 to -1 ms, or the post-stimulus period of 1 to 2000 ms, and within-trial electrodes were individually rejected if activity exceeded ± 100 µV during the same pre- and post-stimulus time periods. Visual analysis of the averaged waveforms indicated that some electrodes became disconnected during recording and were replaced with the mean of their nearest neighboring electrodes. The late positive potential (LPP) component was defined as the mean positive deflection occurring between 500 and 1000 ms post-stimulus, translated into bins of the 128 Hz downsampled signal. Visual inspection of this component revealed that the LPP’s maximal amplitude was broadly distributed around CPZ. Based on this, a cluster consisting of fourteen electrodes (C1, C2, C3, C4, CPZ, CP1, CP2, CP3, CP4, PZ, P1, P2, P3, and P4) were averaged for further analysis.

### Proposed physiological measurement

To assess EEG and ERP brain activity as participants engaged in the built environment, we employed approaches which have been previously validated. These include conventional time-domain measures (simple window averages, 0–500 ms and 500–1000 ms), as well as more advanced time–frequency amplitude and phase-synchrony approaches (the latter indexing functional connectivity between brain regions). These methods are detailed briefly below.

#### Time–frequency (TF) amplitude measures of theta and delta

Our work with these methods has demonstrated that TF approaches can separate overlapping processes during conventional time-domain event-related potential (ERP) components (particularly during the 0–500 ms after stimulus presentation). We have developed a TF approach^4^ that differentiates theta (3–7 Hz) and delta (0–3 Hz) contributions to common ERP components, such as the P3^42,43^, feedback negativity (FN)^44–46^, error-related negativity (ERN)^41,47^, and response inhibition during no-go responses in a go/no-go task^48^. This work indicates that the neurodynamics of many common ERP measures can be understood as a mixture of separate processes occurring simultaneously in theta and delta frequency ranges that are obscured in the unfiltered signal.

Measures for this study include theta and delta in 0–500 ms and 500–1000 ms time windows. Original signals were bandpass filtered separately for delta (0–3 Hz) and theta (3–7 Hz), the RID TF transform^6^ was then applied, and the activity indexed within the defined windows. Delta is often associated with deep sleep and mediation, while theta is associated with deep mediation, and is our gateway to learning, memory, and intution. In theta, our senses are withdrawn from the external world and focus on signals originating from within. High theta and delta activities indicate less mind wandering.

#### TF interchannel phase synchrony (ICPS): indexing functional connectivity

In a previous study, our group developed and validated a measure of functional connectivity for EEG/ERP data based on TF interchannel phase-synchrony (ICPS)^42^, and it was used in this study. We have demonstrated that the data is sensitive to medial-frontal to lateral PFC functional integration underlying cognitive control processes as commonly assessed with the ERN^49–51^, no-go^52^, and FN^53,56^. Measures for ICPS followed the same windows at the TF amplitude measures, taking the average phase-synchrony within the windows instead of amplitude.

## Results and findings: CB versus SB

Results were presented from the still images and video; based on the TF amplitude and ICPS approaches explained in Sect. 4.5, we chose to investigate TF amplitude and functional connectivity (FC) in relation to hypotheses 1 and 2. The TF amplitude analysis provided additional information about neural synchrony that was not apparent in the ongoing EEG. It reveals which brain wave frequencies have the most power at specific points in time and space and how their phase angles synchronize across time and space^54^. FC is defined by measuring similarities between brain signals arising from two regions. Studies show that a person’s creativity capacity can be reliably predicated from the strength of FC^55,56^.

### Still images

**Figure 6:**
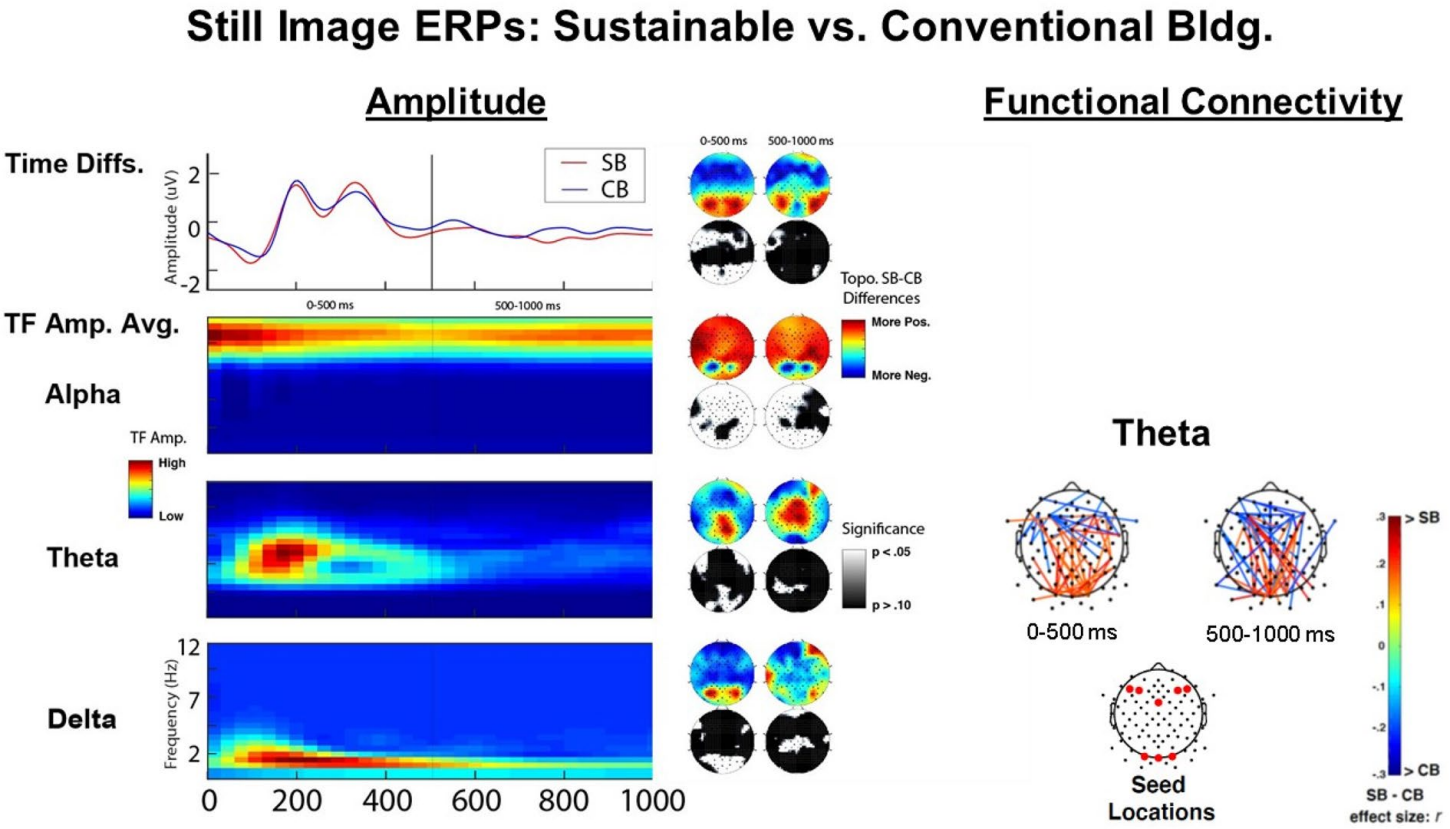
Still image ERPs: sustainable versus conventional building.

Results from the still image presentation are shown in Fig. 6 and demonstrate robust differences between the SB and CB designs. The left panel presents the amplitude effects and the right theta band frontal and occipital functional connectivity (described further below). For amplitude, the top row contains traditional time-domain activity, and the three rows below that depict the time–frequency activity for alpha (8–12 Hz), theta (3–7 Hz), and delta (0–3 Hz). The topo maps to the right of the amplitude plots depict the amplitude differences (color topo plots, where red indicates relatively greater amplitude for the SB, and blue indicates relatively greater amplitude for the CB) and the associated significance (black and white topo plots, where white indicates p < 0.01, and black indicates p > 0.10, uncorrected Wilcoxon nonparametric comparisons). The first column presents average activity across the earlier 0–500 ms time range, and the second column the later 500–1000 ms range.

The time-domain amplitude results in the 0–500 ms range depict significantly greater activity in bilateral occipital regions (associated with processing visual information) and broad decreases in frontal regions (associated with control processing, problem-solving, movement, and social interaction^57^). Time-domain activity in the 500–1000 ms range does not show clear differences. TF results decompose this activity, as well as indexing activity not observable in the time-domain and indicate significant differences between the SB and CB designs for each of the measured frequency bands. Occipital processing increases for the SB, relative to the CB, are readily apparent for each band in the 0–500 ms range (delta, theta, and alpha), suggesting increased engagement in occipital areas for multiple processing systems. It is important to note that alpha activity plays an inhibitory role, and thus decreases in alpha for SB (blue color, for SB-CB, i.e., greater alpha amplitude for the CB) areas were associated with increased occipital processing for the SB. This increase in occipital processing is sustained in the 500–1000 ms range only for alpha. Next, broad and bilateral increases in frontal alpha were observed in the 0–500 ms range, continuing through 500–1000 ms, with bias towards the left side during this later period. Again, increases in alpha are generally associated with greater inhibition of activity. Finally, relatively localized increases in lateral-frontal areas can be observed in the theta band, where hypotheses predicted modulated engagement of lateral prefrontal areas associated with control.

Functional connectivity results for the theta band are presented in the right side of Fig. 6. The hypotheses centered on modulated engagement within medial and lateral frontal regions, linked to control processing, as well as within occipital regions associated with visual processing, as well as long-range communication between these regions. Seed (reference) sites are depicted in a separate topo plot below the results (i.e., significant activity between these sites and all other sites is depicted). Lines represent significant interchannel phase-synchrony between sites (p < 0.05, uncorrected), with effect sizes (*r*-values from the Wilcoxon comparisons) represented by the color (red indicating increases for the SB, and blue increases for the CB). Effects here are consistent with time and TF effects, indicating decreased connectivity within frontal regions, increased activity within occipital regions, and increases between bilateral occipital and bilateral frontal see sites – for the SB relative to the CB. Significant effects were also observed for delta and alpha bands but were not easily interpreted relative to hypotheses during this pilot work. Further treatment of these effects will be undertaken when additional data is collected and assessed. Potential inferences from these findings will be discussed in Sect. 6.0.

### Video

**Figure 7:**
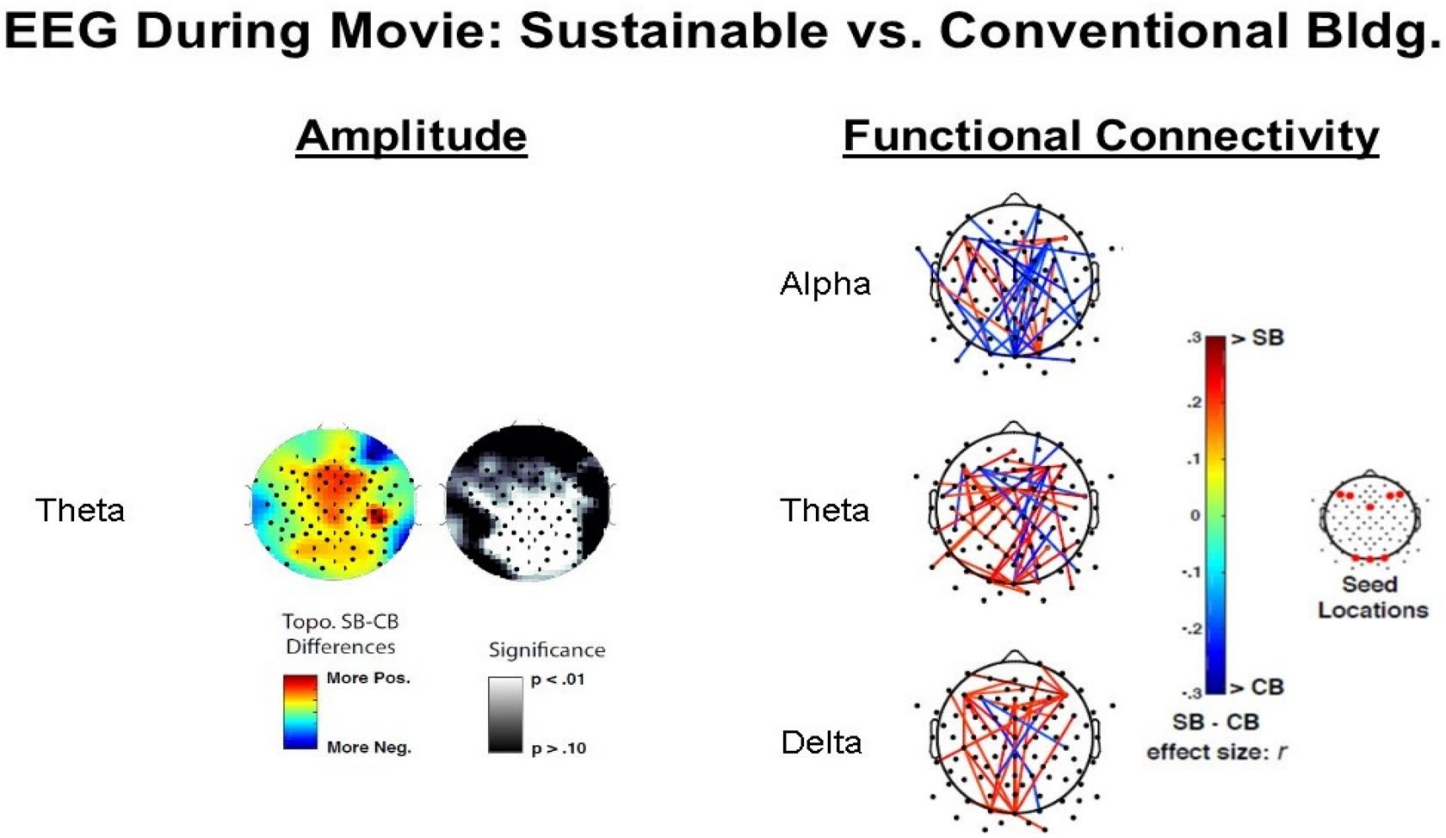
EEG during the movie: sustainable versus conventional building.

Results for the continuous EEG recorded during the movie are presented in Fig. 7. Amplitude results, in the left part of the figure, indicate significant increases in theta band activity in the SB, including medial-frontal, centroparietal, and occipital areas. Differences were not observed in alpha or delta frequency bands. For the functional connectivity (TF-ICPS measures), significant modulations were observed in the SB. Delta and theta evidenced substantially more significant increases (red lines) for the SB relative to the CB, while alpha evidenced substantially more significant decreases (blue lines) for the SB relative to the CB. These effects are consistent with the idea of theta and delta as excitatory (increases for the SB relative to the CB) and alpha as inhibitory (decreases for the SB relative to the CB). The indication of these findings will be discussed in the following section, 6.0.

Overall, the recorded data provided evidence supporting differences between SB and CB features. For hypothesis 1, test subjects demonstrated increased visual system engagement in the SB compared to that in the CB. For hypothesis 2, test subjects exhibited increased modulated attentional focus and control processing in the SB compared to that in the CB.

## Discussion and conclusion

The purpose of the present study is to develop an empirically driven approach to investigating the “soft” benefits of SBs using cognitive-neuroscience methods. Based on the initial empirical data, the primary findings are detailed and explained. This is part of a larger research project that seeks to further understand the psychological and cognitive impact of SBs. The present study provides initial validation of the proposed approach of combining electroencephalography (EEG) and event-related potentials (ERPs) with images and videos from emerging virtual design technology approaches to characterize cognitive-affective processing relevant to occupant experiences in proposed built environments. This sets the stage for planned extensions to using virtual design approaches with EEG/ERP in immersive virtual reality (VR) environments. Initial findings suggest that SB built environments may encourage a shift, consistent with mindfulness, toward a more active and engaged mental stance, with a greater focus on the present environment relative to internal mental processing. The contributions of this study can be discussed at two levels: the cognitive-affective processing modulated by SBs, and the method validation.

### Cognitive-load processing modulated by SBs

The present findings can be explained by the cognitive load theory, which is consistent with the interpretation of greater focus on the present environment and reduced internal mental processing (cf. mindfulness), based on the observed increased theta/delta activities and greater engagement of visual systems and corresponding decreases in frontal activity in the SB environment. Emerging empirical work suggests that exposure to natural environments increases mindfulness^4,5,58^ (e.g., greater engagement with the present environment), supporting the inference that integration of views of nature, and possibly greater spatial views of the interior of the built environment, are central to the observed effects. Mindfulness has been associated with receptive attention and perceptual clarity^59^. Further, improvements in mindfulness have demonstrated reductions in workplace burnout and perceived stress, as well as improvements in personal well-being and team and organizational performance and climate^60^.

One way to conceptualize this shift is in terms of reductions in task-unrelated mental processing (cf. mind wandering), which has been closely investigated in empirical research on mindfulness^61,62^. This may be particularly relevant for the observed effects in the alpha band, which represent inhibitory processes. Here, focused reductions over occipital regions, combined with broad increases in alpha across other regions, are consistent with decreases in a mode of mind wandering, described as “sensory decoupling” or “attentional decoupling.” That is, reductions in a mental state that is dominated by self-referential thought and is relatively disconnected with present-moment environmental circumstances^63–65^. The present findings of reduced bilateral frontal theta-band connectivity in the SB environment, relative to CBs, further supports the notion of a more mindful mental state, as hyper connectivity between frontal regions has been associated with low mindfulness and heightened mind wandering^66^. Work in this area documents how mind wandering is associated with reduced productivity^67,68^, mental health, and mindfulness^34,69^. Reflected as less mind wandering, in an SB environment, the extrinsic cognitive load is lower, which gives the brain more power to process the intrinsic load. Since the intrinsic load is directly related to the ability of processing and solving the given cognitive tasks, such as creating, reading, and comprehending, the occupant could produce better solutions. In addition, in an SB environment, there are fewer cognitive distractions, which typically refers to the amount of cognitive resources demanded from the building occupant by a competing activity. In the case of mind wandering, it is a type of competing activity that can distract people’s attention from other tasks^70^.

The idea of increased occupant mindfulness as a mechanism is consistent with the reports that occupants in SB environments feel stronger environmental satisfaction and support^71^. Physical workplace satisfaction has been positively associated with job satisfaction and better performance, and environmental satisfaction has been linked to contributions to the well-being of residents^72^, particularly the elderly^73^.

### Method validation

Regarding the ability to manipulate and isolate the environmental stimuli, three built environment variables were used to elicit the brain activation and response. This was achieved by using virtual design technologies to change the views, lights, and spatial arrangement of the SB, compared to the CB. As shown in Figs. 6 and 7, the data indicates a clear difference in how subjects responded to different environments, and the clear difference in subjects’ responses to still images versus a more realistic three-dimensional environment (video). The detailed manipulation and assessment of the EEG/ERP measures has the potential to offer new insights into the specific elements most impactful to occupants’ cognitive and affective processing and well-being, The present findings suggest that a cognitive-neuroscience empirical assessment based on a virtual design process can provide a cost-effective approach to basic science efforts to parameterize core beneficial features of SBs, as well the effects of manipulating identified features in specific proposed built environments, before beginning costly construction. These virtual design technologies provide methods for detailed and systematic manipulation of environmental parameters, which would be prohibitively expensive to study in real environments. Such a method provides opportunities for designers to change the design based on the neuroscience response to the preferable built environment, hence identifying the optimized design solution of the built environment.

### Contributions of this study

The validated system and method can be applied in a virtual environment with a simulated built environment; therefore, the design team will be able to simulate the occupant’s response to the design, then revise, modify, and improve related design parameters based on the response. Even though people spend the majority of their time indoors, the role of architecture in shaping human experience is still not well understood^74^. There are some studies focusing on the connection between physical environment and humans’ cognitive function^75^. For example, Heerwagen found the visual connection to daylight through the interior partition (glazing) increased satisfaction in officer workers even if they did not have direct daylight in their immediate space^76^. Sternberg and team demonstrated spatial familiarity and predictability are key to the safety and autonomy of seniors, so when designing a hospital room for elderly patients, the designer should put more consideration into the location of the entry door and the process by which the patient moves across the open space to a desired point^77^. However, the existing literature connecting the cognitive function’s impact on design is limited. The findings of studies provide observations and are not in-depth enough to help provide an understanding of the reasons that can be utilized to improve the design of physical environment^44^. For example, they observed that children in classrooms lit with natural daylight achieve higher test scores but could not determine why this happens. Meanwhile, quantitative scientists, including neuroscientists, often see architectural design as a profession mainly concerned with aesthetic beauty. Building design is more than aesthetics; expanding the horizon for neuroscience would eventually result in a new knowledge base for building design, particularly sustainable building design, since the impacts of sustainable building features often needs to be quantified.

### Limitations and next steps

This study has several limitations that are important to note. First, while several building parameters were identified (e.g., lighting, spatial dimensions, and nature views), these were all assessed together. As the impact of buildings on occupants is a cumulative process in which all building parameters provide contributions, it will be more beneficial and useful to understand more about those parameters’ influences and impacts, respectively. In planned future research, we will isolate the effect of each design parameter for assessment. The analysis and empirical data on individual parameters can then be translated into design guidelines or techniques. Similarly, the different spaces (eg., private versus public) in the assessed SB were assessed together, making inferences about parameters of specific types of spaces (e.g., open areas, group rooms, individual offices) difficult to infer. Future work should separately manipulate and assess different types of spaces. Additionally, one primary built environment was assessed, so it is not possible to infer which manipulated parameters may be more general versus those that may be more tied to a specific proposed built environment. The last important limitation is related to the long-term effects of building environment. In the virtual environment we only measured the short-term effects, but in reality, the buildings will have a long-term impact on people’s cognition and neurophysiological changes. It is unknown whether the short-term changes induced in the virtual environment can be representative of the long-term real impact. In the next steps, we will need to evaluate to what extent the experimental results in the virtual environment can be used to index the effects in the real condition.

Despite some limitations, the present study makes several valuable contributions to better understanding the impacts of SBs. First, the core approach provided robust differences between SB and CB virtual environments, with readily interpretable findings (e.g., mindfulness), providing validation that the proposed cognitive-neuroscience/virtual-design approach can index important differences in the occupant experience of the built environment. These preliminary results thus serve as a foundation to direct future research steps, since understanding the correlation between brain activity and environmental stimuli could help us to identify the environmental-related causal factors of cognitive performance^15^. Several future steps we plan to take in this direction are:

- Use a larger number of participants; currently, we are planning to collect data sets from 200 additional testing subjects. This will allow several advances:
  - Increased power to make detailed inferences about underlying mechanisms
  - Possibility to assess individual differences
  - Independent manipulation of proposed design parameters, to isolate contributions from each parameter
- Integrate a full virtual reality (VR) period, in addition to still images and movies:
  - Assess effects in a more immersive environment, which has been shown to enhance effects^78^
  - Direct cognitive assessment tests in the immersive VR environment, to simulate task performance
- Create a multiuser VR immersion:
  - Multiple test subjects will be immersed in the same environment, providing opportunities to assess interactions with others and increasing collaborative productivity.

## Abbreviations

ERP: Event-related potential
VR: Virtual reality
EEG: Electroencephalogram
SB: Sustainable building
CB: Conventional building
CLT: Cognitive load theory
ICPS: Interchannel phase synchrony
TF: Time–frequency
